# Genomic and Phenotypic Divergence in Wild Barley Driven by Microgeographic Adaptation

**DOI:** 10.1002/advs.202000709

**Published:** 2020-11-13

**Authors:** Jianxin Bian, Licao Cui, Xiaoyu Wang, Guang Yang, Fulin Huo, Hubin Ling, Liqin Chen, Kuijun She, Xianghong Du, Boaz Levi, Adi Jonas Levi, Zhaogui Yan, Xiaojun Nie, Song Weining

**Affiliations:** ^1^ State Key Laboratory of Crop Stress Biology in Arid Areas College of Agronomy and Yangling Branch of China Wheat Improvement Center Northwest A&F University Yangling Shaanxi 712100 China; ^2^ College of Life Science Jiangxi Agricultural University Nanchang Jiangxi 330045 China; ^3^ Reidman College Tel Aviv 6997536 Israel; ^4^ Faculty of Sciences and Technology Tel Hai College Upper Galilee 1220800 Israel; ^5^ College of Horticulture and Forestry Sciences Huazhong Agricultural University Wuhan 430070 China; ^6^ Australia‐China Joint Research Centre for Abiotic and Biotic Stress Management in Agriculture Horticulture and Forestry Yangling Shaanxi 712100 China

**Keywords:** adaptive evolution, edaphic adaptation, genetic diversity, whole genome resequencing, wild barley

## Abstract

Microgeographic adaptation is a fundamental driving force of evolution, but the underlying causes remain undetermined. Here, the phenotypic, genomic and transcriptomic variations of two wild barley populations collected from sharply divergent and adjacent micro‐geographic sites to identify candidate genes associated with edaphic local adaptation are investigated. Common garden and reciprocal transplant studies show that large phenotypic differentiation and local adaptation to soils occur between these populations. Genetic, phylogenetic and admixture analyses based on population resequencing show that significant genetic divergences occur between basalt and chalk populations. These divergences are consistent with the phenotypic variations observed in the field. Genome sweep analyses reveal 162.7 Mb of selected regions driven by edaphic local adaptation, in which 445 genes identified, including genes associated with root architecture, metal transport/detoxification, and ABA signaling. When the phenotypic, genomic and transcriptomic data are combined, HvMOR, encoding an LBD transcription factor, is determined to be the vital candidate for regulating the root architecture to adapt to edaphic conditions at the microgeographic scale. This study provides new insights into the genetic basis of edaphic adaptation and demonstrates that edaphic factors may contribute to the evolution and speciation of barley.

## Graphical abstract

Massive variations are observed on the phenotypic traits, genomic diversity, and transcriptomic dynamics of the two wild barley populations collected from sharply divergent and adjacent microgeographic sites. HvMOR was found to regulate the root architecture of wild barley to adapt to basalt and chalk edaphic conditions. This study sheds light on the genetic mechanism of microgeographic adaption and genomic divergence associated sympatric speciation in barley and beyond.

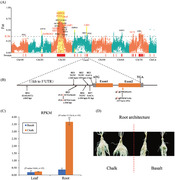

## 1 Introduction

Evolution is the most fundamental biological process and is essential for all organisms living in a dynamic world. Although the general process of evolution is supported by diverse lines of evidence, the underlying interplay of molecular changes and population dynamics often remains obscure.^[^ ^1^ ^]^ Evolutionary divergence driven by adaptation to the environment originally proposed by Charles Darwin (1859) was gradually confirmed to be one of the key forces of speciation. Heterogeneous environments generally exert strong selection pressures on the species inhabiting them to drive adaptive diversification of populations even across short distances on the landscape, which is called as microgeographic adaptation.^[^ ^2^ ^]^ Microgeographic adaptation is considered as the equilibrium between selection pressures and the homogenizing effects of gene flow; this adaptation occurs when a population shows higher fitness in its native environment than in a foreign environment, representing a mechanism that favors population differentiation and subsequently leads to eventual evolution, even speciation.^[^ ^3^ ^]^ Among all organisms, plants provide a unique opportunity to study the development and evolutionary process of local adaptation to the environment and resource availability because they are rooted, with their sessile characteristics and evolution dynamics mainly affected by edaphic conditions. Once the continuity of physical and chemical properties is disrupt, divergence in phenotypic traits can be observed.^[^ ^4^ ^]^ Extensive studies have indicated that, following generation continuity and heredity stabilization, genetic diversity and speciation can increase, even under microgeographic conditions.^[^ ^5^ ^]^ It has been reported that changing both the mating system and flowering time can result in a high degree of prezygotic genetic isolation among closely adjacent metal‐tolerant and non‐tolerant populations of the sweet grass (*Anthoxanthum odoratum)*.^[^ ^6^ ^]^ In *Collinsia sparsiflora*, hybrid sterility over tens of meters caused by the strong adaptive differentiation was observed between adjacent and very recently diverged lineages.^[^ ^7^ ^]^ Differences in flowering time and genetic loci were also observed in palms, a finding that was consistent with models of sympatric speciation involving disruptive/divergent selection.^[^ ^8^ ^]^ With the emergence of population genomic sequencing, an increasing number of studies have been performed with respect to the “genomic islands of speciation” metaphor,^[^ ^9^, ^10^ ^]^ which hypothesizes that either a few isolated chromosomal regions or many loci distributed throughout the genome can drive speciation through gene flow.^[^ ^11^, ^12^ ^]^

The Alma‐Har‐Ben‐Zimra site is located in the eastern region of Upper Galilee, Israel (33.03°E, 35.29°N), with a typical Mediterranean climate.^[^ ^13^ ^]^ The soils on chalk and basaltic rocks here exhibited significant divergence from each other in term of their physical and chemical properties as well as soil texture.^[^ ^10^ ^]^ Basalt soil is clayey, has a soft texture, is rich in organic matter and has good moisture and fertilizer conservation. In contrast, chalk soil is sandy, with a hard texture and low porosity, but it is poor in organic matter, water and nutrient resources.^[^ ^14^ ^]^ Noticeably, different ecosystems have arisen in basalt and chalk soils due to their differences in resource availability.^[^ ^10^ ^]^ These sharply adjacent but divergent basalt and chalk ecologies provide an ideal system to study the microgeographic adaptation. By the use of the blind mole rats inhabiting in these two soils as a model, large‐scale studies have been conducted to investigate adaptive evolution based on mtDNA,^[^ ^10^ ^]^ AFLPs,^[^ ^15^ ^]^ whole‐genome resequencing^[^ ^11^ ^]^ and transcriptomic data,^[^ ^16^ ^]^ providing evidence to support the theory of sympatric speciation (SS) at the molecular level. Similar studies with molecular markers have also been performed in wild barley (*Hordeum vulgare*) and wild emmer wheat (*Triticum turgidumL. var. dicoccoides)*,^[^ ^10^ ^]^ but little information is available for in‐depth studies at the whole genome level combined with genetic and phenotypic investigations such as reciprocal planting to identify the genomic divergence underlying phenotypic counterparts, potentially eventually leading to speciation.

To better understand the mechanism of edaphic natural selection on plants at the genomic level, we conducted whole genome resequencing of 18 wild barley accessions from basalt microsite and 15 from Chalk microsite to analyze their genetic diversity, genomic divergence, and population structure. Furthermore, the genomic regions associated with edaphic adaptation were identified through selection sweep analysis, and candidate genes involved in these processes were obtained by integrating population genomic, transcriptomic and phenotypic traits.

## 2 Results

### 2.1 Chemical Composition of Basalt and Chalk Soils at the Alma‐Har‐Ben‐Zimra Site

**Figure 1:**
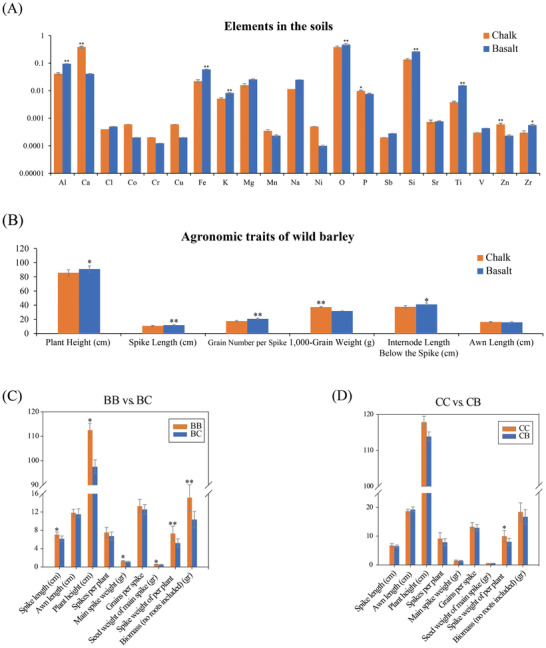
Comparison of the phenotypic values of basalt and chalk wild barley populations and the edaphic composition of basalt and chalk soils. A) Elements in basalt and chalk soils. B) Agronomic performance of basalt and chalk wild barley during a common garden study. C,D) Phenotypic values from the reciprocal transplant experiments. C) Phenotypic value and plasticity of plants of the basalt wild barley population growing in basalt soil (BB) and chalk soil (BC). D) Phenotypic value and plasticity of plants of the chalk wild barley population growing in chalk soil (CC) and basalt soil (CB). *,*p*< 0.05; **,*p*< 0.01.

As described previously, the soil on basaltic rock at this site (basalt soil) has a dark reddish‐brown appearance while the soil on chalk rock (chalk soil) appears pale‐brown to whitish.^[^ ^10^, ^14^ ^]^ After examining their chemical compositions, we found that these two neighboring soil patches encompass significantly different profiles of inorganic substances from each other even though they are adjacent. In chalk soil, Ca^2+^ (38.41%) was the most abundant, followed by Si^4+^ (13.52%), Al^3+^ (4.14%), Fe^3+^ (2.23%), Mg^2+^ (1.60%) and other elements (**Figure** **1**A). Compared to that in the chalk soil, the composition of elemental content in the basalt soil was obviously different, with Si^4+^ being the most abundant (25.54%), followed by Al^3+^ (9.54%), Fe^3+^ (5.91%) and Ca^2+^ (4.09%) (Figure 1A). Through statistical analysis, the elemental contents of Al^3+^ (*p* = 9.51E‐05), Fe^3+^ (*p* = 0.00048), Si^4+^ (*p* = 0.00075), Ti^4+^ (*p* = 2.07E‐06) and Ca^2+^ (*p* = 0.00020) showed significant differences between the basalt and chalk soils (Figure 1A), suggesting that different resource availability and/or stresses could be exerted on plants inhabiting these two microenvironments, as these elements play critically important roles during the whole lives of plants. Particularly, the percentage of Ca^2+^ in the chalk soil (38.41%) was ≈8.39‐fold higher than that in the basalt soil (4.09%), which may result in a specific calcium homeostasis mechanism that evolved in chalk wild barley population for their survival in this specific environment compared with that in the adjacent basalt population.

### 2.2 Phenotypic Variations in the Wild Barley Population Inhabiting Basalt and Chalk Soils

Common garden and reciprocal transplant experiments were conducted to detect the phenotypic evidence for genetic differentiation and local adaptation of wild barley to basalt and chalk edaphic condition (Figure 1B–D and Figure S1, Supporting Information). The phenotypic traits of basalt and chalk wild barley populations grown in a common garden were first investigated and compared. The average plant height and the spike length of basalt wild barley were 91.01 ± 8.88 cm and 11.68 ± 1.14 cm respectively, while those of chalk wild barley were 85.74 ± 5.99 cm and 10.73 ± 0.99 cm, respectively. Compared with the chalk population, the basalt population had significantly greater plant height and longer spike length (*p* = 0.011 and 0.002). Moreover, the average internode length below the spike of the basalt plants was also notably higher than that of the chalk plants, with the value of 41.09 ± 4.99 cm and 37.60 ± 5.63 cm (*p* = 0.025), respectively. Furthermore, the average number of grains per spike of the basalt wild barely was 20.53 ± 1.58, which was also remarkedly higher than that of the chalk wild barley, with an average of 17.52 ± 1.15 (*p* = 0.008). Interestingly, the 1000 grain weight of the chalk population was significantly greater than that of the basalt population, with an average of 37.00 ± 2.52 g for the chalk plants compared to 31.4 ± 3.82 g for the basalt plants (Figure 1B), which was due to the drier conditions and then lower wild barley population density in chalk soil compared with the wetter condition and higher wild barley population density in the basalt soil.^[^ ^10^, ^14^ ^]^ Plants of the basalt and chalk populations were collected from adjacent regions that had almost identical environmental conditions except for sharply divergent soil conditions. Edaphic factors play a crucial role in plant growth and development, as plants are rooted in soil to obtain essential water and nutrients. Therefore, the extensive phenotypic trait variations of wild barley from the basalt and chalk microsites provided effective evidence that genetic differentiation has been shaped in basalt and chalk wild barley populations.

To explore the local adaptation and genetic fitness of wild barley to basalt and chalk soils, a reciprocal transplanting study was performed (Figure 1C,D). The reciprocal trials were carried out in Upper Galilee, northern Israel (33.23°N, 35.58°E), which has similar Mediterranean climate similar to that of the site where the wild barley accessions were collected. The results showed that the basalt wild barley displayed better fitness in the basalt soil, whereas the chalk wild barley exhibited better fitness in the chalk soil (Figure S1, Supporting Information). When basalt wild barley accessions were grown in the less fertile chalk soils, they had significantly shorter main spike lengths, lower plant height, and lower whole‐spike weights and aboveground biomass compared to those of basalt wild barley grown in its native basalt soil (Figure 1C). These findings suggest that basalt soil can provide much richer water and nutrient resources for wild barley than can chalk soil, as the wild barley on the basalt soil showed better phenotypic values. On the other hand, when chalk wild barley accessions were planted in the more fertile basalt soil with better water retention ability, they did not show better agronomic performances compared to that of chalk wild barley grown on the chalk soil. In contrast, the phenotypic values of the chalk wild barley grown on chalk soil were higher than those of the chalk wild barley grown on basalt soil for all the recorded traits (Figure 1D), even though the whole biomass of the chalk wild barley grown on chalk soil was significantly higher than that of basalt wild barley grown on basalt soil (Figure S1, Supporting Information). It has been demonstrated that when populations develop a higher fitness in their native environment compared with foreign environments, a local adaptation between them forms.^[^ ^2^, ^17^ ^]^ Therefore, the basalt and chalk wild barley accessions appear to have developed local adaptations to their specific soil microenvironments.

### 2.3 Massive Genetic Divergence between These Two Wild Barley Populations

**Figure 2:**
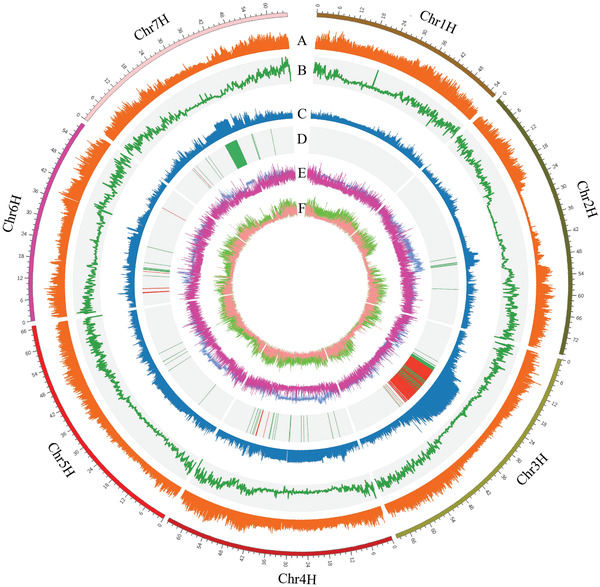
Summary of resequencing data of 33 wild barley plants. A) SNP density, B) INDEL density, C) Fst, D) Selected sweep regions (red bar, basalt; green bar, chalk), E) Tajimas’ D‐basalt (blue), Tajimas’ D‐chalk (purple), F) Pi‐basalt (light green), Pi‐chalk (light red).

Whole genome resequencing of the 33 wild barley accessions generated a total of 1.11 Tb of data, with an average of 33.6 Gb and corresponding to ≈7 × depth for each individual (Table S1, Supporting Information). After aligning the reads to the reference genome and stringent quality filtering, a total of 3 379 287 INDELs (shorter than 50) and 57 238 496 high‐quality SNPs were identified, representing the largest wild barley variation dataset to date (**Figure** **2**). All the filtered SNPs were then annotated, and most of the high‐quality SNPs were located in intergenic regions (72.68%), followed by intron (12.51%) regions; only 2.56% of these SNPs were exonic (**Table** **1**). Within the coding regions, a total of 1 150 198 nonsynonymous SNPs and 717 351 synonymous SNPs were observed, with a non‐synonymous to synonymous ratio of 1.49. The numbers of heterozygous and homozygous INDELs and SNPs were also calculated (Figure S2A,B and Table S2, Supporting Information). The results showed that the mean Het levels were significantly higher in the basalt population than in the chalk population; conversely, the mean Hom of the chalk population was higher than that of the basalt population (Figure S2, Supporting Information).

**Table 1:** Distribution of SNPs within various genomic regions of 33 wild barleys

Variants type	SNP count	Percent
Total number	75 798 553	1
Intergenic	55 090 986	72.68%
Intronic	9 483 543	12.51%
Downstream	3 420 597	4.51%
Upstream	3 400 762	4.49%
Exonic	1 938 374	2.56%
UTR_3_PRIME	1 333 542	1.76%
UTR_5_PRIME	922 847	1.22%
Synonymous	717 351	0.95%
Missense_variant	1 150 198	1.52%
Splice_region_variant	207 901	0.27%
Stop gain	70 878	0.09%
Stop loss	5061	0.01%

**Figure 3:**
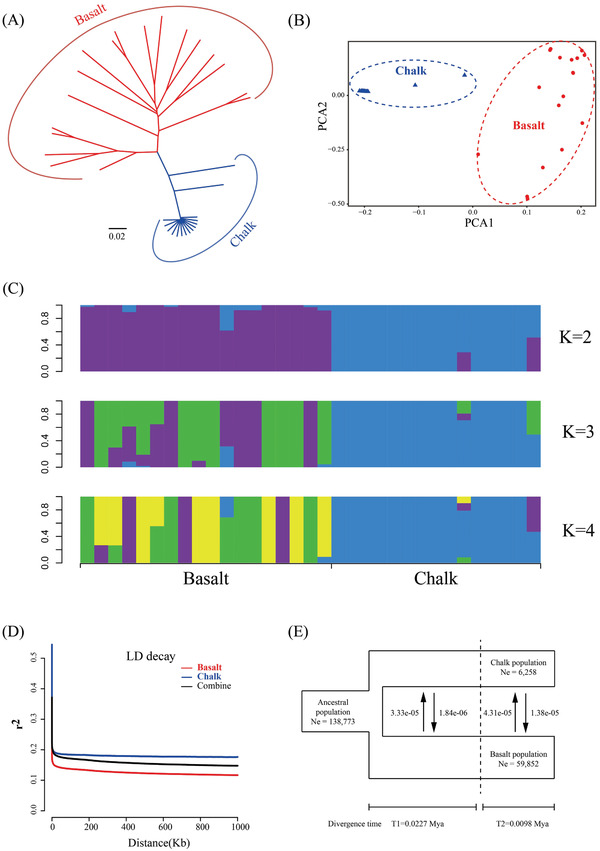
Genetic diversity and population divergence of 33 wild barley accessions. A) Neighbor‐joining phylogenetic tree based on all of the SNPs. Red, basalt individuals; blue, chalk individuals. The reliability of each branch was evaluated by bootstrapping with 1000 replicates. B) Principal component analysis of these wild barley plants. The first principal component clearly separated the two populations. C) Population structure of two wild barley populations when*K*= 2, 3, and 4. D) LD decay of the two wild barley populations. The x axis stands for physical distances (bp), whereas the*y*axis stands for*r* ^2^values. E)*δ*a*δ*i analysis showing the demographic history of basalt and chalk wild barley population. They were split by a single divergence event with two different stages (*T*_1_≅ 0.0227 Mya and*T*_2_≅ 0.0098 Mya, respectively). Effective population size (Ne) remained unchanged across the two divergence stages, while different migration rate during (*T*_1_) and after (*T*_2_) last glacial maximum (LGM). The average number of migrants between them is shown between the black arrows.

To reveal the genetic relationship between the basalt and chalk wild barley populations, we first constructed a neighbor‐joining (NJ) tree to examine the relationship of the two populations. The tree clearly grouped all of the basalt accessions into one cluster, while all of the chalk accessions were grouped into another cluster, suggesting that obvious genetic divergence occurred between them (**Figure** **3**A). Principal component analysis (PCA) also revealed that the accessions collected from the same soil clustered together, and were obviously separated from the individuals of the other soil type (Figure 3B). To further estimate ancestry proportions of each accession, population structure was evaluated based on *K* = 2, 3, and 4 (Figure 3C), and the results showed that the optimal *K* value was 2 for these 33 barley accessions. Although a few accessions showed a mixed genetic background when *K* = 2, the basalt population was completely separated from the chalk population, which was consistent with the PCA and NJ‐tree results. Moreover, with *K* = 3 and 4, accessions from basalt population were further split into two and three groups, and six accessions (B3, B4, B5, B6, B9, B11) showed admixed ancestry, while the chalk accessions (except C10 and C15 accessions) maintained their homogeneous genetic background, probably due to the lower population density that caused by the more heavily stressed environment. This result indicated that ongoing gene flow was present in these two adjacent wild barley populations, although significant genetic divergence has still occurred.

Furthermore, the population‐based genetic diversity (*θπ*) of the basalt population (mean *π* = 2.90 × 10 ^−3^) was 2.48‐fold higher than that of the chalk population (mean *π* = 1.17 × 10 ^−3^) (Figure 2F), indicating that approximately half of the genetic diversity was lost in the chalk population compared to the basalt population. Additionally, linkage disequilibrium (LD; indicated by *r* ^2^) decreased to half of its maximum value within 12 kb for the two populations (Figure 3D). The higher linkage disequilibrium level in the chalk population reflected relatively higher inbreeding and fewer recombination events in harsher environments, which were consistent with the population structure and genetic diversity analysis.

To elucidate the evolutionary scenario that might explain the obvious difference between the basalt and chalk population, we inferred the demographic history of the two barley populations using the diffusion approximation for demographic inference (*δ*a*δ*i) approach.^[^ ^18^ ^]^ The best‐fitting model (“asym_mig_twoepoch”) showed that there was a single divergence event with two different stages among the two populations; one stage is at ≈0.0227 Mya and the other is at ≈0.0098 Mya (Figure 3E). Effective population size (Ne) was estimated at 59 852 and 6258 for the basalt and chalk populations, respectively, and remained unchanged across the two divergence stages. Additionally, it is obvious that continuous asymmetric migration occurred with different migration rate at T1 and T2 stages. The migration rates of basalt to chalk and chalk to basalt were 3.33E‐05 and 1.84E‐06 at T1, and 4.31E‐05 and 1.38E‐05 at T2, respectively, suggesting that gene flow occurred continuously between them (Figure 3E).

### 2.4 Genome Regions and Genes Associated with Edaphic Natural Selection

To identify potential mechanisms governing microgeographic adaptation, genomic regions affected by soil selection were inferred by Wright's F‐statistic (Fst) and Tajimas’ D analysis between the two wild barley populations, with a window size of 100 kb.^[^ ^11^ ^]^ Based on the observed distribution of the two statistics, we identified candidates of positively selected genomic regions as those with Fst values above the 90th percentile (Figure 2C) and Tajima's D values within the 5th percentile (Figure 2E). In total, 1238 and 389 candidate regions under selection were identified, harboring 384 and 94 genes for the basalt and chalk populations, respectively (Figure 2D and Table S3, Supporting Information). The major selected regions (80%) were located in chromosome 3H, and followed by chromosome 7H (11.37%) and the remaining regions (8.63%) were located on other chromosomes (Figure 2E). The genes under the selective sweep were then classified into functional categories by Gene Ontology (GO) and Kyoto Encyclopedia of Genes and Genomes (KEGG) enrichment analyses. Selected genes in basalt population were significantly enriched in “proton transmembrane transporter activity” (GO:0015078, 15 genes, *p* = 6.64E‐06), “ion transmembrane transporter activity” (GO:0015075, 25 genes, *p* = 2.51E‐05), “monovalent inorganic cation transmembrane transporter activity” (GO:0015077, 16 genes, *p* = 5.17E‐05), “transport” (GO:0006810, 65 genes, *p* = 6.87E‐06), “ATP metabolic process” (GO:0046034, 13 genes, *p* = 6.02E‐04), “ATP synthesis coupled proton transport” (GO:0015986, 7 genes, *p* = 8.68E‐04) (Table S3, Supporting Information), and oxidative phosphorylation pathway (26 genes, *p* = 1.48E‐11) as well as photosynthesis (8 genes, *P* = 0.002382496) (Figure S3 and Table S3, Supporting Information). For the chalk population, selected genes were significantly enriched in “peptide biosynthetic process” (GO:0043043, 9 genes, *p* = 4.35E‐04), “macromolecule biosynthetic process” (GO:0009059, 14 genes, *p* = 1.78E‐03) and “cellular nitrogen compound biosynthetic process” (GO:0044271, 14 genes, *p* = 2.19E‐03) (Figure S3A,B, Table S3, Supporting Information). KEGG enrichment analysis showed that the selected genes were significantly enriched in the “oxidative phosphorylation” (11 genes, *p* = 7.73E‐7), “phagosome” (7 genes, *p* = 3.45E‐05), and “carbon metabolism” (7 genes, *p* = 0.025) pathways in the basalt population (Figure 3C, Table S3, Supporting Information), and the chalk population was significantly enriched in the “photosynthesis” (3 genes, *p* = 1.04E‐3), “metabolic pathways” (13 genes, *p* = 4.99E‐03), “sulfur metabolism” (2 genes, *p* = 5.34E‐03) and “cysteine and methionine metabolism” (2 genes, *p* = 0.037) pathways (Figure S3C,D and Table S3, Supporting Information). We then integrated the selected genes with the phenotypic variations and edaphic conditions to identify the candidate genes associated with micro‐geographic selection and adaptation. Two genes involved in the calmodulin‐binding (HORVU3Hr1G049640 and HORVU3Hr1G052770) were identified, which could contribute to the local adaptation to high‐calcium stress in the chalk population. One genes (HORVU3Hr1G040360) related to citrate metabolism and one gene in the heavy metal transport/detoxification superfamily (HORVU3Hr1G049780) were also found (Table S3, Supporting Information), which are crucial for metal transport/detoxification. Furthermore, many drought‐responsive and ABA signaling related genes were enriched in various sweep regions, such as stomatal cytokinesis defective (SCD1) (HORVU3Hr1G050010), ABA‐responsive protein‐related (HORVU3Hr1G039680), and a Cytochrome P450 family gene (HORVU3Hr1G056770) (Table S3, Supporting Information), suggesting that the contrasting moisture conditions between basalt and chalk soil drive the genetic divergence of these two wild barley populations. Interestingly, the identified genes associated with drought adaptation were significantly enriched in 3H, indicating that 3H is the vital genomic region target for discovering drought‐tolerant genes and is involved the molecular mechanism of drought tolerance and adaptation.

To explore the genetic divergence of the two wild barley populations shaped by the soil micro‐environment, we analyzed the haplotype of six selected genes related to early flowering gene (HORVU3Hr1G048610), Ca^2+^ transport or signaling (HORVU3Hr1G052770), auxin signaling response factor (HORVU3Hr1G049860), metal transport/detoxification (HORVU3Hr1G052420), Cytochrome P450 (HORVU3Hr1G056770) and tonoplast‐localized half‐size ABC transporter (HORVU6Hr1G021320). Overall, the results showed that the two wild barley populations present dramatically different haplotype organizations (Figure S4 and Table S4, Supporting Information). Compared with chalk population, the basalt population showed much higher genetic diversity and more haplotypes. For the HORVU3Hr1G048610 (EF3), HORVU3Hr1G052770 (CML16), HORVU3Hr1G052420 (ALR1), and HORVU6Hr1G021320 (ALS1), there are 3, 3, 4, and 4 haplotypes, respectively, in the basalt population, and only 2, 2, 1 and 1 haplotypes, respectively, in the chalk population. These results further demonstrated that the obvious genetic divergence was shaped by the sharply contrasting edaphic conditions.

### 2.5 Transcriptomic Variations between Basalt and Chalk Wild Barley Populations

**Figure 4:**
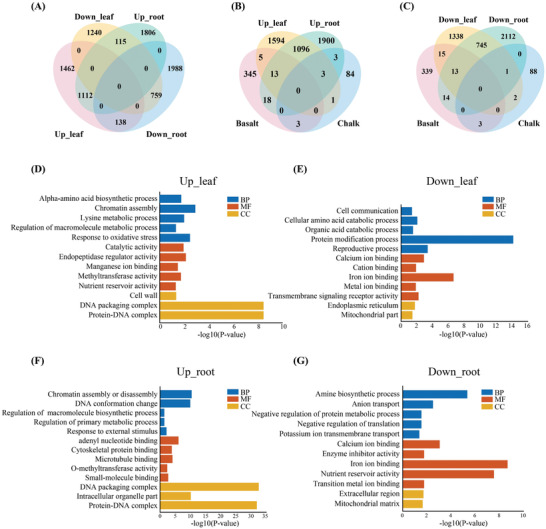
Venn diagrams and GO enrichment of DEGs in wild barley. A) Overlapping and unique differentially expressed genes (DEGs) in the leaf and root organs of basalt and chalk accessions. B) Overlap of upregulated differentially expressed genes (DEGs) and select genes in the basalt and chalk populations. C) Overlap of downregulated differentially expressed genes (DEGs) and select genes in basalt and chalk populations. Upleaf, upregulated in the leaves; Downleaf, downregulated in the leaves; Uproot, upregulated in the roots; Downroot, downregulated in the roots. D–G) GO enrichment of differentially expressed genes in the leaves and roots.

To identify the key adaptive genes, population transcriptome analysis was performed. Root and leaf samples collected from 5 basalt and 5 chalk accessions (3 biological replications each) were subjected to RNA sequencing. A total of 3.95 billion raw reads (593.20 Gb) for the 60 samples of the two wild barley populations were obtained. Like the phenotypic and genomic variations, there was also massive transcriptomic variation between the basalt and chalk barley populations (**Figure** **4**). In total, 4826 and 5918 differentially expressed genes (DEGs) were identified between the leaves and roots of the two populations, respectively, of which 2712 and 3033 genes were upregulated in the leaves and roots of basalt accessions respectively, while 2114 and 2885 showed higher expression in the leaves and roots of chalk accessions, respectively (Figure 4A, Tables S5 and S6, Supporting Information). In addition, 1112 DEGs overlapped between the leaf and root samples in the basalt accessions, while only 759 overlapping genes were found in the chalk accessions. To integrate the DEGs and selected genes, 16 and 14 selected genes showing differential expression between the leaves and roots, respectively (Figure 4B,C, Tables S7–S12, Supporting Information). GO enrichment analysis of the DEGs showed relatively high expression of genes significantly enriched in 73 and 96 GO terms in the leaves and roots of the chalk accessions, respectively, while the highly expressed genes in the leaves and roots of the basalt accessions were enriched in 105 and 157 GO terms, respectively (Tables S13–S16, Supporting Information). Notably, 33 (*p* = 0.0013) and 48 (*p* = 0.00087) genes related to calcium ion binding (GO:0005509) terms were uniquely found among the significantly highly expressed genes in the leaves and roots of the chalk accessions (Tables S14 and S16, Supporting Information), which was consistent with the significantly higher Ca^2+^ content in the chalk soil than basalt soil. Furthermore, 66 (*p* = 2.40E‐07) and 93 (*p* = 2.10E‐09) genes related to iron ion binding (only the GO:0005506 term was found) in the leaf and root organs of the chalk accessions (Tables S14 and S16, Supporting Information), together with 8 genes (*p* = 0.042) related to potassium ion transmembrane transport (GO:0071805), were also uniquely identified in the roots of the chalk accessions (Table S16, Supporting Information), suggesting that wild barley inhabiting chalk soil evolved the ability to locally adapt to the chalk soil conditions where iron and potassium ions are scarce but Ph is high. In addition, upregulated genes in the chalk barley were also found to be uniquely enriched in terms associated with cellular amide metabolic processes (GO:0034249), negative regulation of cellular protein metabolic processes (GO:0032269), negative regulation of protein metabolic processes (GO:0051248) and negative regulation of translation (GO:0017148); conversely, upregulated genes in the basalt population were uniquely enriched in terms associated with regulation of biosynthetic process (GO:0009889), regulation of cellular biosynthetic process (GO:0031326), regulation of nitrogen compound metabolic process (GO:0051171) and regulation of primary metabolic process (GO:0080090) (Tables S13 and S15, Supporting Information). KEGG enrichment analysis revealed that in the roots, upregulated genes in the basalt population were significantly enriched in pathways related to ABC transporters, base excision repair, and wax biosynthesis while those in the chalk population were enriched in pathways related to carbon fixation in photosynthetic organisms, galactose metabolism and plant hormone signal transduction. On the other hand, in the leaves, the upregulated genes of basalt barley were significantly enriched in pathways related to the circadian rhythm, glycerolipid metabolism and plant hormone signal transduction, but those of in chalk barley were enriched in arginine and proline metabolism, biosynthesis of amino acids, cutin, suberine, and wax biosynthesis, and cysteine and methionine metabolism (Figure S5, Supporting Information). These results demonstrated that the differential gene expression patterns and divergent transcriptome dynamics between the basalt and chalk populations were shaped by adjacent but sharply contrasting soil conditions.

### 2.6 Identification of the Key Genes Regulating Edaphic Adaptation

**Figure 5:**
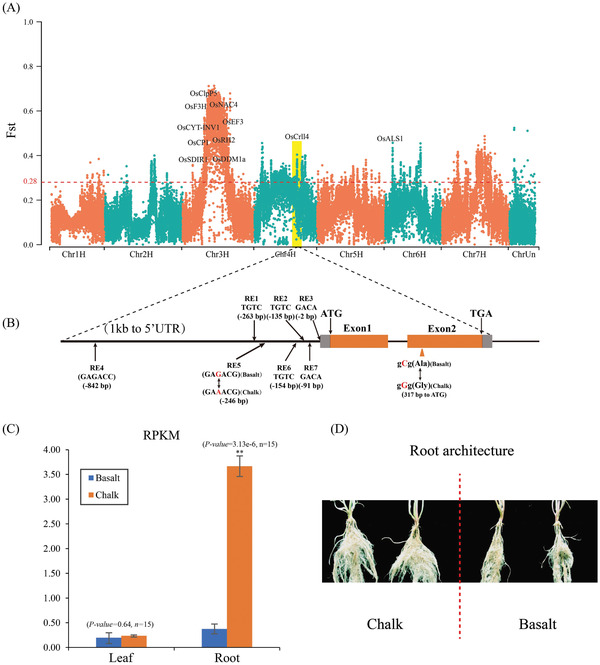
HORVU4Hr1G056890 is the vital adaptive gene regulating root architecture for local adaptation. A) Homologous rice genes of selected genes between the basalt population and chalk population. B) Structure of HORVU4Hr1G056890. Orange rectangles, ORFs; gray rectangles, 5′ and 3′ UTRs; orange arrow, nonsynonymous nucleotide substitution in the basalt population compared with the chalk population; RE1, RE2, RE3, RE4, RE5, RE6, and RE7, AuxREs in the promoter region of HORVU4Hr1G056890 (the numbers in parentheses show the position of each element relative to the start of the 5′ UTR). C) Analysis of RPKMs of roots and leaves between basalt and chalk populations (blue, basalt; orange, chalk). D) Difference in root architecture of the basalt and chalk wild barley populations.

Combining genome sweep and gene expression analysis, we found that the selected gene HORVU4Hr1G056890 showed significantly differential expression between the roots of the plants in the chalk and basalt populations (**Figure** **5**A,C, Table S11, Supporting Information), with 10 times higher expression in the chalk than in the basalt population. HORVU4Hr1G056890 was annotated as a LOB (LATERAL ORGAN BOUNDARIES) domain‐containing protein (LBD), and it is an orthologous gene of *TaMOR*, the more roots (MOR) gene from wheat. A previous study has reported that overexpression of *TaMOR* significantly improved the number of lateral roots in Arabidopsis, caused larger root systems as well as increased grain yields of rice.^[^ ^19^ ^]^ The much higher expression of HORVU4Hr1G056890 could regulate the chalk population to form the more lateral roots and larger root systems compared to those of the basalt population (Figure 5D). To validate it, we investigated and compared the root related traits between basalt and chalk barley accessions. Result found that the chalk population showed larger and more lateral roots, while the basalt population had smaller and fewer lateral roots (Figure 5D). Furthermore, the root surface area and root length in basalt wild barely were significantly higher than that of chalk wild barley, while the root diameter and root tip number were significantly lower in basalt compared to chalk population. We then investigated the haplotype of HORVU4Hr1G056890 in the basalt and chalk wild barley populations and then associated the root related traits with haplotype. In total, 2 SNPs were found in this gene. All of the basalt wild barley accessions showed identical haplotypes (haplotype I) that differed from the haplotype observed in all wild barley accessions from the chalk soil (haplotype II) (Figure 5B and Figure S6A, Supporting Information). One SNP (C to G) occurring at the coding region position 317 from the ATG was identified in all of the chalk wild barley accessions, resulting in the codon gCg (Gly) changing into gGg (Ala). It has been demonstrated that the same mutation at the 317 position of TaMOR in wheat resulted in the improvement of root architecture and drought tolerance of wheat.^[^ ^19^ ^]^ The other SNP (G to A) was found in the promoter region of HORVU4Hr1G056890 in all chalk accessions, while no SNP was found in any basalt accession. Further analysis showed that this SNP was located in the CGTCTC motif (RE5), which is an important cis‐element that binds to auxin to induce the expression of LBD genes and then influence root initiation.^[^ ^20^ ^]^ It is postulated that this SNP could affect the binding strength of ARF and then induce the differential expression of the MOR gene. Association of root‐related traits with these two haplotypes indicated that they had significant differential effects on root traits. The root surface area, root length, root diameter and root number of haplotype I (basalt) were significantly higher than those of haplotype II (chalk), which was consistent with the results of wheat^[^ ^19^ ^]^ (Figure S6, Supporting Information). In conclusion, the sequence variation of HORVU4Hr1G056890 could regulate the chalk wild barley to have more lateral roots and increased root surface area to enhance the capacity of water absorption and nutrient acquisition due to the shallow and stressful soil in chalk microsite. HORVU4Hr1G056890 (HvMOR) is considered a crucial adaptive gene that regulates the root architecture to adapt to the basalt and chalk edaphic conditions.

## 3 Discussion

The concept of sympatric speciation (SS) has been highly controversial since it was first advocated by Darwin.^[^ ^21^ ^]^ Interestingly, increasing numbers of studies have revealed that the closely related taxon adaptation to diverse environments, genetic differentiation and sympatric speciation can occur even within a microsite with the continual gene flow. Overall, an important premise for sympatric speciation involves the microgeographic adaptation and genetic differentiation. The wild barley population collected from the sharply divergent and adjacent basalt and chalk soils serves as an ideal model system to study sympatric speciation. Here, a total of 33 wild barley accessions (18 accessions from the basalt site and 15 accessions from the chalk site) were used as materials. We first investigated and compared a series of phenotypic traits between them through common garden and reciprocal transplant experiments. Significant phenotypic differentiation between them was found, and both of them showed better fitness in their natural environment than in foreign environments, suggesting that the local adaptation of phenotypes to the basalt and chalk microenvironment has occurred. This provided evidence about microgeographic adaptation from the perspective of morphological and agronomic traits. Population resequencing was then performed to reveal the genetic differentiation and the underlying basis of local adaptation. Based on the large SNP dataset, the basalt and chalk populations displayed two distinctly divergent groups, and the basalt population showed higher genetic diversity than did the chalk population. PCA and population structure analysis also revealed the distinctly different result between the two wild barley populations. These results suggested that the genomic divergence occurred in the two wild barley populations from basalt and chalk soils, although they are adjacent (less than 100 m). Admixture accessions were then observed following the increase in the assumed ancestor number (*K* = 3, 4, and 5) in the basalt and chalk populations, suggesting that gene flow and hybridization between the two populations are still ongoing, with the migration of seeds possibly mediated by ants, rodents, and other vectors.^[^ ^22^ ^]^ A previous study reported the similar results for the basalt and chalk wild barley population by allozyme analysis.^[^ ^22^ ^]^ Wang et al also found that the obvious genetic divergence occurred in wild barley populations in basalt and terra rossa soil types by whole‐genome resequencing.^[^ ^23^ ^]^ More importantly, the genetic diversity of the basalt population was 2.48‐fold higher than that of the chalk population. Therefore, significant variations in genetic diversity between the basalt and chalk populations are comparable to those of the wild types versus domesticated types in several of ephemeral or perennial crop species, such as barley,^[^ ^24^ ^]^ soybean,^[^ ^25^ ^]^ and apple.^[^ ^26^ ^]^ These findings were completely opposite to those observed for blind mole rat, as the chalk population showed higher genetic diversity than did the basalt population.^[^ ^11^ ^]^ This may be due to the rooted characteristics of plants, as they cannot move to choose a suitable environment and must adapt to soil conditions. Soil conditions exert more impact on plants than on animals. Thus, plants grown under abutting but contrasting soil conditions provide the valuable insight into natural selection and speciation in evolution.^[^ ^4^ ^]^

Furthermore, a significant difference in the composition of elements was found between the basalt and chalk soil, particularly for Ca^2+^, Al ^3+^, and Fe^2+^. Based on the genome sweep analysis, a series of genes related to Ca^2+^ transport or signal transduction were identified as genes that play important roles in cellular Ca^2+^ homeostasis (Table S3, Supporting Information). A gene that was found in the selected regions and that is related to citrate synthase in the basalt population, may be crucial for reducing aluminum toxicity and the consequence of adaptation to high Al^3+^ stress (Table S3, Supporting Information). Additionally, the higher calcareous edaphic conditions and lower Fe content in the chalk soil than in the basalt soil may have allowed evolution of an effective approach for an Fe absorption mechanism to meet the requirements of normal growth, and several Fe absorption and transport related genes were found in the selected regions. The moisture of basalt soil is higher than that of chalk soil, which may result in the difference in water availability and drought tolerance of the two wild barley populations. When drought stress leads to decreased water availability, ABA is significantly increased to stimulate stomatal closure, alter gene expression, and regulate adaptive physiological responses to improve the water use efficiency and drought tolerance.^[^ ^27^ ^]^ We identified a series of genes related to ABA synthesis, accumulation and signal transduction as well as some osmotic adjustment‐related genes, which are tightly associated with drought adaptation (Table S3, Supporting Information). These results supported that edaphic conditions exerted strong selection on wild barley, and the identified selection signatures in the genome provide a vital resource for mining candidate genes for environmental adaptation.

Phenotypic investigation revealed that the basalt and chalk wild barley populations showed entirely different root architectures that the chalk plants displayed larger and more lateral roots while the basalt plants exhibited smaller and less lateral roots. It is well known that root system plays vital roles in the normal growth of plants, such as water absorption, nutrient acquisition, anchorage, propagation, storage functions, secondary metabolite synthesis, and accumulation.^[^ ^28^ ^]^ The significantly differential root architecture reflected the adaptation to the basalt and chalk soils. To compete for limited water and nutrient resources, wild barley in the chalk soil has evolved an efficient root architecture for survival. By integrating resequencing and RNA‐seq analysis, we found that one selected gene HORVU4Hr1G056890 showed significant differential expression between the roots of the basalt and chalk wild barley populations and the amino acid variations were significantly associated with the diverse root architecture, indicating that the phenotypic divergence and genetic differentiation occurred between them at the genic level and that HORVU4Hr1G056890 was the vital adaptive gene regulating this process.

In summary, soil composition played a key role in shaping the genetic divergence of two abutting wild barley populations and the plants in these populations have also evolved the efficient adaptive mechanisms to cope with soil stress. The selected genomic regions and candidate genes associated with edaphic adaptation will not only contribute to a better understanding of the evolutionary process but also provide useful information to identify genes for genetic improvement of barley.

## 4 Experimental Section

##### Plant Materials and Phenotype Investigations

The seeds of wild barley were collected from the sharply contrasting but adjacent chalk and basalt microsites in northern Israel (33.03°E, 35.29°N) in an expedition in spring, 2013. The seeds of 18 accessions (named basalt 1 to basalt 18) were collected from the basalt soil, and the seeds of 15 accessions (named chalk 1 to chalk 15) were collected from the chalk soil. A common garden and reciprocal transplant study were then conducted to investigate the local adaptation and genetic fitness of wild barley to the chalk and basalt soils. For the common garden study, all of wild barley accessions were grown in the field at Northwest A&F University, China (108.08°E, 34.30°N), and their morphological and agronomic traits were examined to detect the genetic differentiation in the 2015–2016 and 2016–2017 cropping seasons, respectively. The reciprocal transplant study was carried out in the 2018–2019 cropping season at Tel Hai College, Upper Galilee, northern Israel (33.23°E, 35.58°N), at a distance of 30 km from the original collection site for wild barley. The seeds of the 18 basalt and 15 chalk accessions were planted in plots with basalt soil (BB, basalt population growing in basalt soil; BC, basalt population growing in chalk soil) or chalk soil (CC, chalk population growing in chalk soil; CB, chalk population growing in basalt soil). A random block design, two and three biological replications for BB, CC and BC, CB were applied, respectively. Three seeds per pot and a single seedling was retained after emergence to prevent competition among seedlings. The pots were watered by drip irrigation and the agronomic traits and phenotypic plasticity of the plants were examined and compared. In addition, the properties of the basalt and chalk soils were also measured and a total of twenty elements (Al_2_O_3_, CaO, ClO, CoO, Cr_2_O_3_, CuO, Fe_2_O_3_, K_2_O, MgO, MnO, Na_2_O, NiO, P_2_O_5_, Sb_2_O_3_, SiO_2_, SrO, TiO_2_, V_2_O_5_, ZnO, ZrO_2_) in the basalt and chalk soil patches were assayed respectively by X‐ray fluorescence (EA1200VX) according to the manufacturer's protocol (Hitachi, Japan).

##### Whole Genome Resequencing and SNP Calling

For whole‐genome resequencing, 15 seeds of each accession were germinated at 4 °C for four nights and then grown at 18 °C for 10 d in a dark growth chamber. Genomic DNA was then extracted using a DNeasy Plant Kit (Qiagen) following the manufacturer's protocol. Afterward, 10 µg of DNA was randomly fragmented and the fragments 500 bp in size were recovered to construct the sequencing library following the manufacturer's protocol. Sequencing was performed on an Illumina HiSeq X‐10 platform in conjunction with PE150 following the manufacturer's protocol. All of the sequence data were deposited in the NCBI Sequence Read Archive (SRA) under accession number of SRP145511. The obtained clean reads were mapped to the reference genome of barley cultivar Morex^[^ ^29^ ^]^ with Burrows‐Wheeler Aligner (BWA) software using the default parameters.^[^ ^30^ ^]^ The mapping results were converted to bam files, and then were sorted after filtering the duplicated reads by SAMtools (v1.1)^[^ ^31^ ^]^ using the “view,” “sort” and “rmdup” parameters. Sequencing coverage and depth of each sample were calculated (Table S1, Supporting Information). Furthermore, population‐based SNPs and INDELs detection of the 33 accessions were performed using a Bayesian approach by SAMtools (v1.1) with the following parameters “mpileup ‐m 2 ‐C 50 ‐t DP ‐t SP ‐t AD ‐F 0.002 ‐q 20 ‐Q 20 ‐d 1000” SNPs and INDELs were then extracted from the population variations as a VCF file. The SNPs were further qualified for downstream analysis using the following criteria: 1) the depth of the variations ranged from 4 to 1000; 2) the unobserved variant allele was > 50%; 3) no gap was present within a 5 bp window; and 4) multinucleotide polymorphisms were ignored. For INDELs, only insertions and deletions shorter than or equal to 50 bp were taken into account. Finally, annotation of the qualified SNPs was performed according to the reference genome using the package SnpEff (v 4_3k).^[^ ^32^ ^]^

##### Phylogenetic and Population Structure Analyses

A neighbor‐joining tree was constructed by TreeBest v1.9.2 using the SNP datasets with a bootstrap value of 1000 and visualized using FigTree (v1.4.3) software. The input bed format was converted from the variant calling format using VCFtools^[^ ^33^ ^]^ and PLINK (v1.90).^[^ ^34^ ^]^ Principal component analysis (PCA) was then conducted using the biallelic SNPs of the 33 individuals by GCTA (v64) software.^[^ ^35^ ^]^ Finally, the population structure was constructed using the ADMIXTURE software.^[^ ^36^ ^]^ To determine the most likely number of ancestral kinships (Ks) among the two populations, we increased the coancestry clusters spanning from 2 to 4 with 10 000 iterations. The lowest *K* value was then obtained, which was used to indicate the most likely number of clusters in the population.

##### Genetic Diversity and LD Analysis

Population‐based genetic diversity (*θπ*) was calculated by VCFtools software. For each population, a sliding window analysis with a window size of 100 kb (‐window‐pi 100 000) and a step size of 10 kb (‐window‐pi‐step 10 000) was used and the mean value of *θπ* was considered the whole genome genetic diversity. Linkage disequilibrium (LD) was calculated based on the correlation coefficient (*r* ^2^) statistics for all pairs of SNPs within 1000 kb using PopLDdecay (v3.29) with the following parameters: ‐MaxDist 1000 ‐MAF 0.05 ‐Het 0.88 ‐Miss 0.25.^[^ ^37^ ^]^ The average *r* ^2^ was then plotted against physical distance using a Perl script (Plot_MutiPop.pl) in PopLDdecay software. The demographic history of the basalt and chalk populations was inferred using *δ*a*δ*i method.^[^ ^18^ ^]^ To ensure neutrality, only the SNPs in the intergenic regions were used and the polymorphic sites were randomly pruned in 5 kb window size to minimize the effects of genetic linkage. All the retained SNPs were then used to calculate the site allele frequencies and construct all the diversification models, with the highest log‐likelihood value considered to be associated with the best‐fitting model. The effective population size, migration rate, and population divergence time were calculated. The divergence time is obtained based on a mutation rate of 6.5E‐09 per generation.

##### Transcriptome Sequencing and Analysis

For RNA‐seq analysis, five basalt and chalk accessions were selected and grown in a controlled growth chamber at 22 °C, with a photoperiod of 18 h/6 h (light/dark), under the same hydroponics conditions. Total RNA was extracted from roots and leaves of three biological replications at the three‐leaf stages (18 d after germination). Purification and quality control were then performed and only the qualified RNA was used for library construction. Illumina reads were filtered by removing the adaptor sequences, empty reads and low‐quality reads (*Q* < 20) with FASTX‐toolkit, and the remaining clean reads were aligned to the barley reference genome using HISAT2 (v2.0.5) with the default parameters.^[^ ^38^ ^]^ All the read count statistics were calculated using HTSeq (v0.7.1), with the “htseq‐count” parameter.^[^ ^39^ ^]^ Gene expression levels were estimated via RPKM values (reads per kilobase transcriptome per million reads). Furthermore, differentially expressed genes were identified using the DESeq2 tool with the parameters padj<0.05 and |log_2_ (fold change)| >1 . Venn diagrams and volcano plots of all the DEGs were constructed via TBtools software.^[^ ^40^ ^]^

##### Selective Sweep Analysis

Tajima's D and fixation statistics (Fst) are informative indicators of population differentiation and selective signatures.^[^ ^11^ ^]^ Fst and Tajima's D values were calculated for a sliding window by VCFtools software, with a window size of 100 kb. The FST and Tajimas’ D values were then sorted in descending order, and the regions with the highest 10% of FST values and the lowest 5% of Tajimas’ D values were considered putative selection regions.^[^ ^41^ ^]^ The genes located in these regions were then considered candidate selected genes. The functional annotations of the candidate genes selected in the genome sweep regions were integrated with the soil element compositions and agronomic traits of barley to identify the key genes associated with edaphic adaptation. The genetic variations (SNPs and INDELs) of these genes were then extracted from the Variant Call Format (VCF) file and compared between the basalt and chalk soil populations. The haplotype of each gene was subsequently determined by DnaSP (v6).^[^ ^42^ ^]^

##### GO and KEGG Enrichment Analysis

GO annotation was conducted using the AgriGO (v2).^[^ ^43^ ^]^ With all the annotated barley (Morex) genes as background, singular enrichment analysis (SEA) via AgriGO was used to detect over‐represented GO categories in each population. GO terms with a corrected FDR less than 0.05 were considered significant. KEGG enrichment was also conducted using KOBAS 3.0 software.^[^ ^44^ ^]^ The *p*‐value less than 0.05 was considered significant and the GO and KEGG enrichment results were plotted using R scripts.

##### Statistics Analysis

All of the statistical analysis was performed using R v3.4.0 software (https://www.r-project.org/). The mean and *p* value was calculated based on the One‐Way ANOVA model. And the standard deviations (SD) were considered as the error line. Significant test was detected at the 0.01 level (*p* < 0.01) labeled with ** and at the 0.05 level (*p* < 0.05) labeled with *.

## Conflict of Interest

The authors declare no conflict of interest.

## Author Contributions

N.X. and S.W. designed and supervised the project. B.J., C.L., and Y.G. collected and generated the data, and performed analysis. H.F. and W.X. performed transcriptome analysis. S.K., L.H., and C.L contributed to data analysis. D.X., L.B., and L.A.J. contributed to plant material collection. B.J. and N.X. prepared the draft manuscript. Y.Z. and S.W. reviewed and revised the manuscript. J.B. and L.C. contributed equally to this work.

## Supporting information

Supporting Information

Supplemental Table 1

## Data Availability

Accession codes. BioProject: Whole genome resequencing and RNA sequencing data have been deposited into Sequence Read Archive (SRA) database in NCBI under accession code PRJNA459495.
