# Supplementary material for: Genomic and Phenotypic Divergence in Wild Barley Driven by Microgeographic Adaptation
Source: Adv Sci (Weinh). 2020 Nov 13;7(24):2000709. doi: 10.1002/advs.202000709 (PMC7740101; doi:10.1002/advs.202000709)
Supplement: Supplementary file 1 — Supporting Information [file ADVS-7-2000709-s001.pdf]

© 2020 Wiley-VCH GmbH

ADVANCED SCIENCE
Open Access

Supporting Information

for *Adv. Sci.,* DOI: 10.1002/advs.202000709

Genomic and Phenotypic Divergence in Wild Barley
Driven by Micro-geographic Adaptation

Jianxin Bian, Licao Cui, Xiaoyu Wang, Guang Yang,
Fulin Huo, Hubin Ling, Liqin Chen, Kuijun She,
Xianghong Du, Boaz Levi, Adi Jonas Levi, Zhaogui Yan, Song Weining, and Xiaojun Nie**

Supplementary Materials

Figure S1. Results of the reciprocal transplant experiment between basalt and chalk populations of wild barley grown in basalt soil and chalk soil. The mean trait values and their changes during the experiment are shown. **, *p*<0.01; *, *p*<0.05.

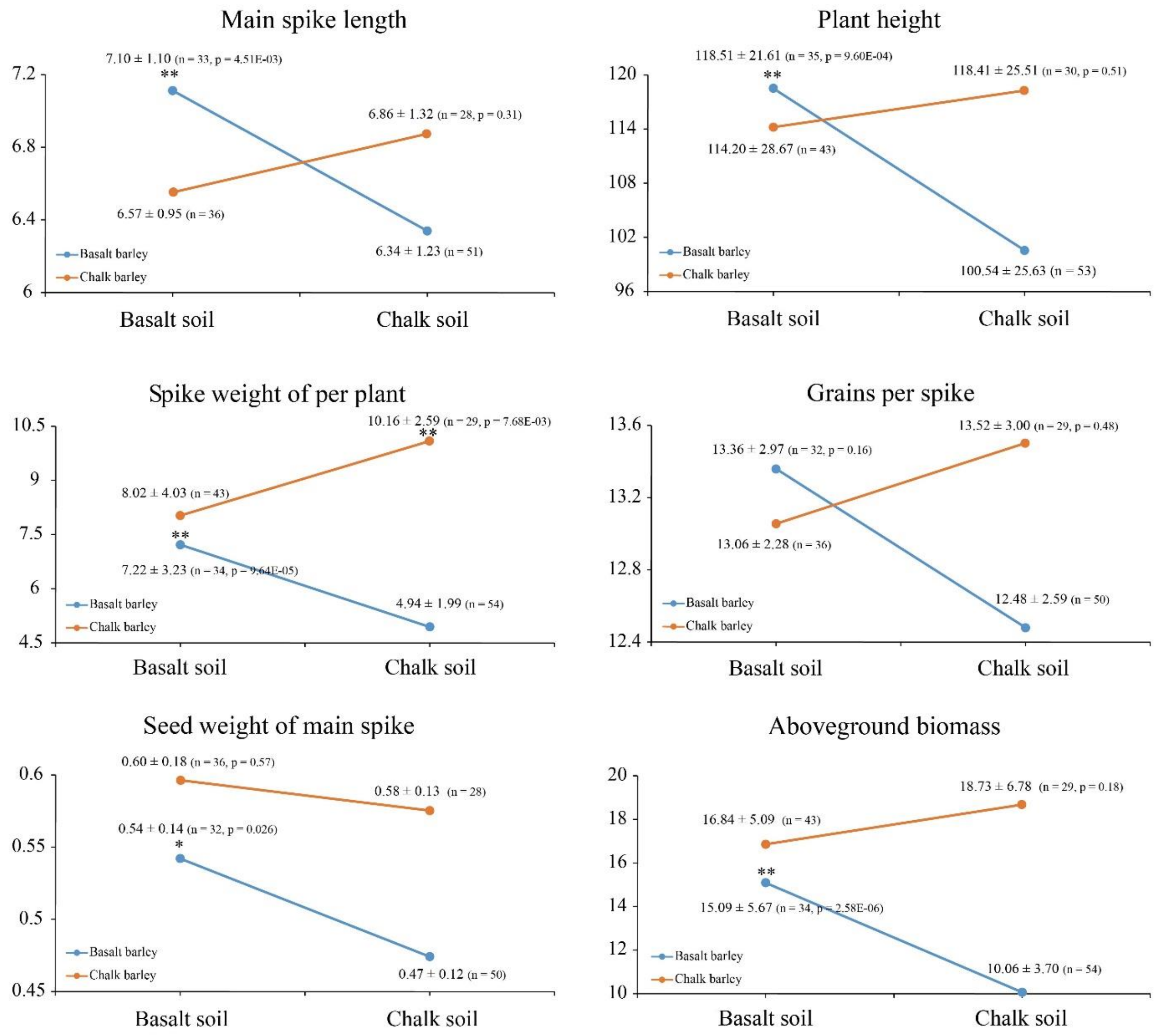

Figure S2. Het/Hom genotypes of SNPs and INDELs. (A) Heterozygous and homozygous genotype analysis of SNPs (Blue, basalt; orange, chalk); (B) Heterozygous and homozygous genotype analysis of INDELs (blue, Basalt; orange, Chalk). **, $p<0.01$; *, $p<0.05$.

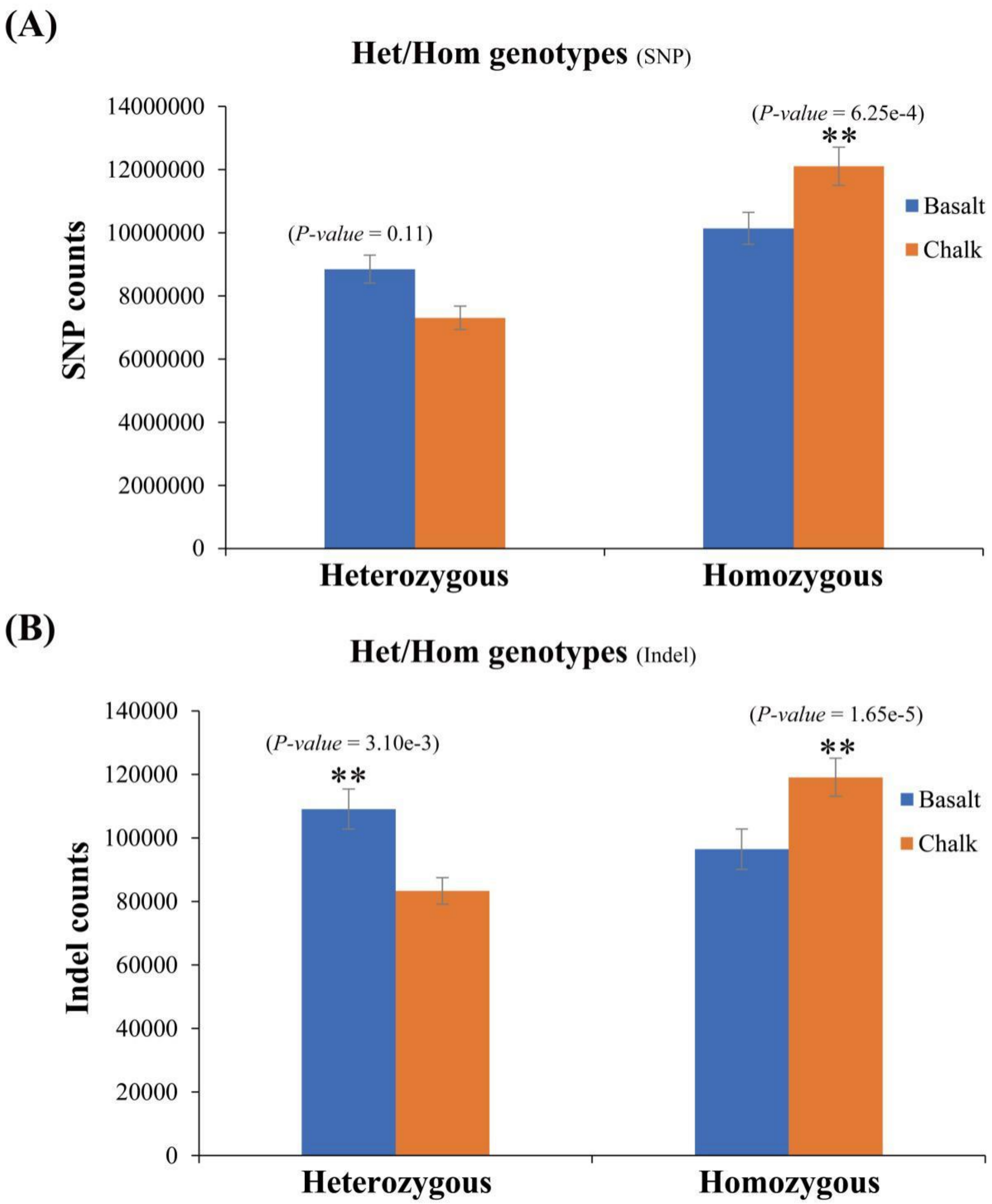

Figure S3. GO and KEGG enrichment of the selected genes in basalt and chalk population. (A) GO enrichment of all the selected genes using AgriGO (v2) with a p-value <0.01 in the basalt population. (B) GO enrichment of all the selected genes using AgriGO (v2) with a p-value <0.01 in the Chalk population. (C) KEGG pathway analysis by KOBAS 3.0 using all the protein sequences of selected genes (P-value<0.05) in the basalt population. (D) KEGG pathway analysis by KOBAS 3.0 using all the protein sequences of selected genes (P-value<0.05) in the chalk population.

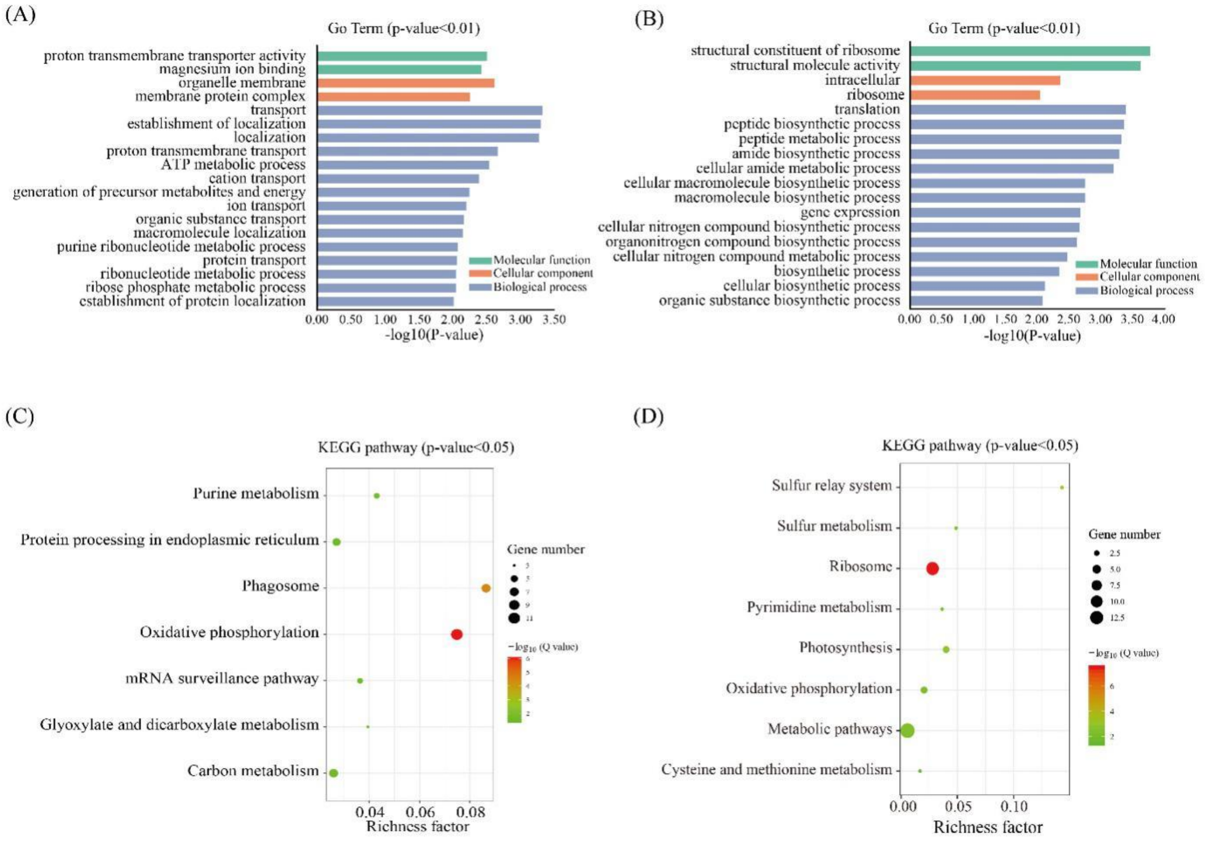

Figure S4. Haplotype and Homology analysis of six selected genes. (1) OsEF3 gene is related to early flowering. (2) OsCrll2 gene is related to auxin signaling response. (3) ALR1 is related to Mg2+ transport. (4) Calcium-binding protein (CML16) are related to signal transduction and Ca2+ transport. (5) CYP97A4 is involved in lutein biosynthesis. (6) OsALS1 gene is related to internal detoxification of aluminum.

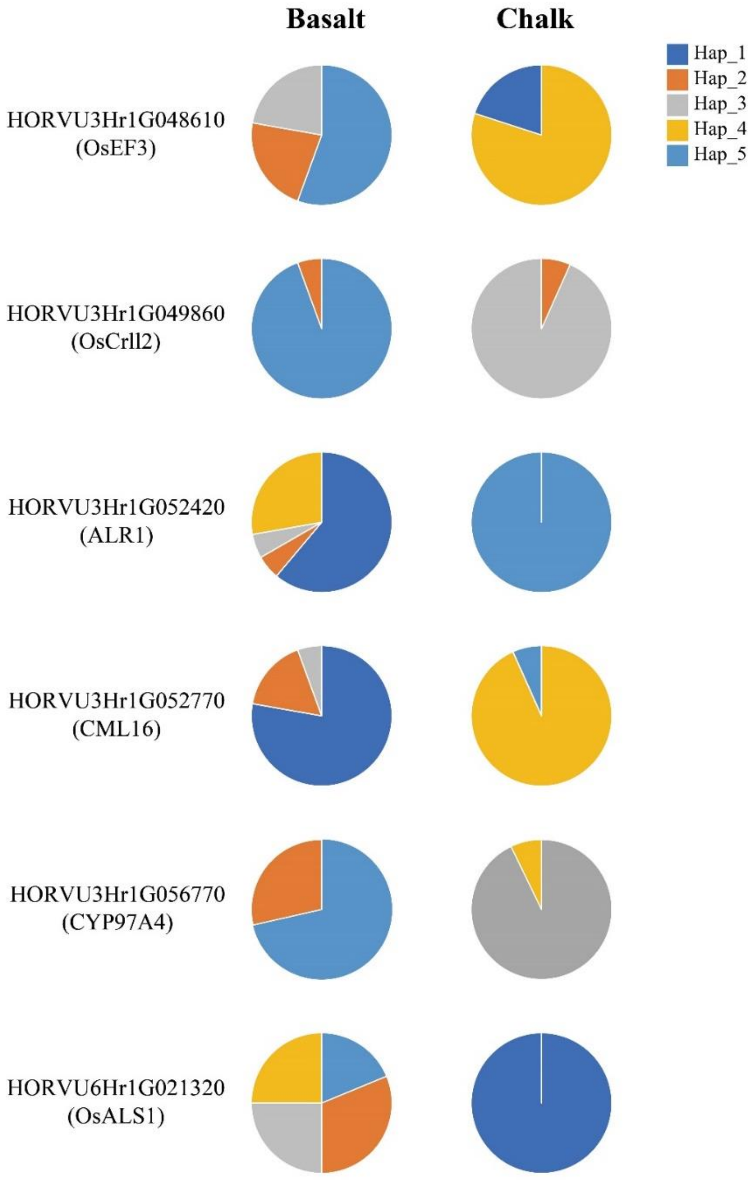

Figure S5. KEGG enrichment analysis of DEGs. (A) KEGG pathway analysis of genes upregulated in the leaves. (B) KEGG pathway analysis of genes downregulated in the leaves. (C) KEGG pathway analysis of genes upregulated in the roots. (D) KEGG pathway analysis of genes downregulated in the roots.

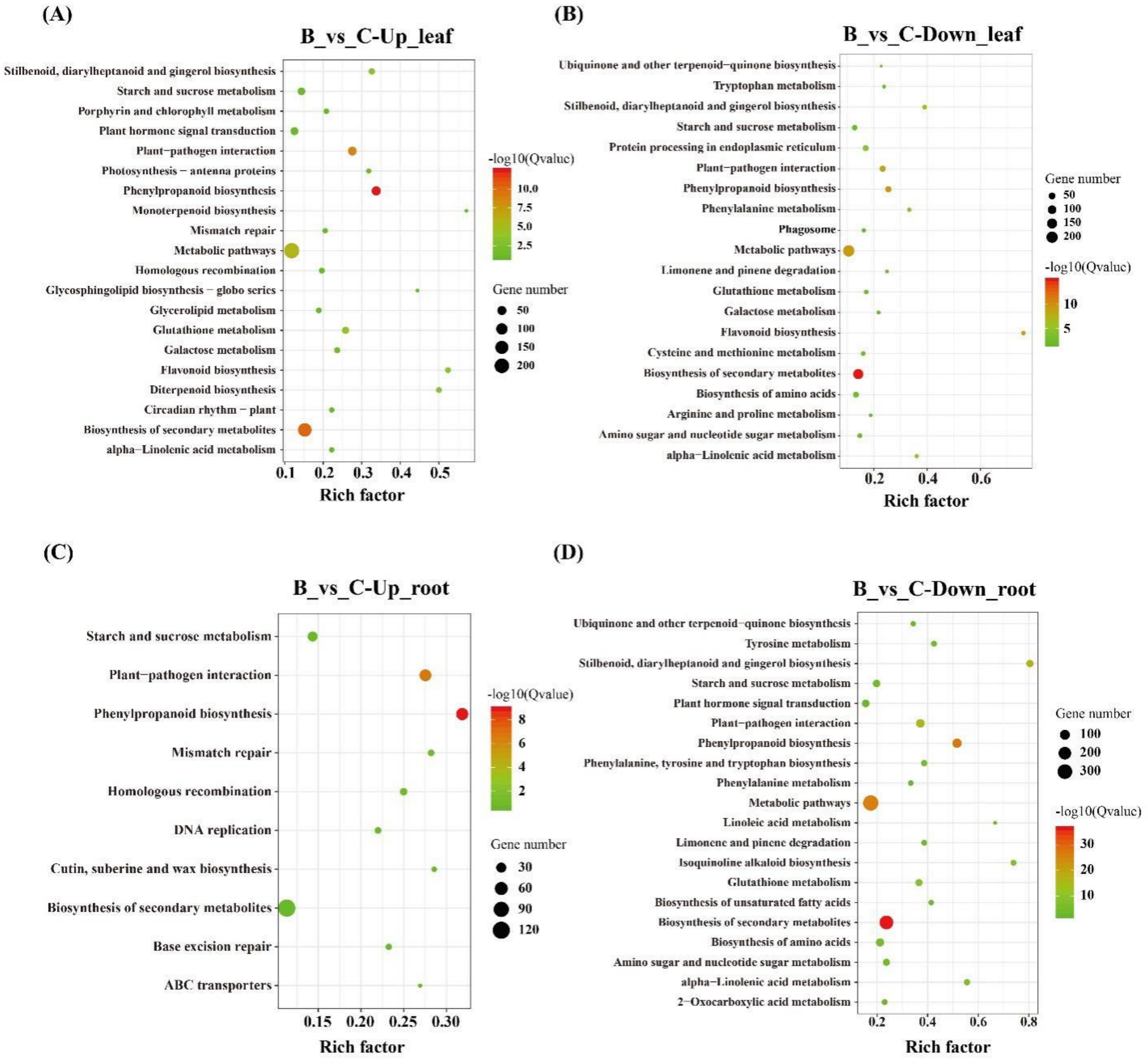

Figure S6. Haplotypes of the HORVU4Hr1G056890 gene and root-related traits in basalt and chalk wild barley. (A) Haplotype I (basalt) and Haplotype II (chalk). (B) Significance analysis of root surface area. (C) Significance analysis of root length. (D) Significance analysis of root diameter. (E) Significance analysis of root number.

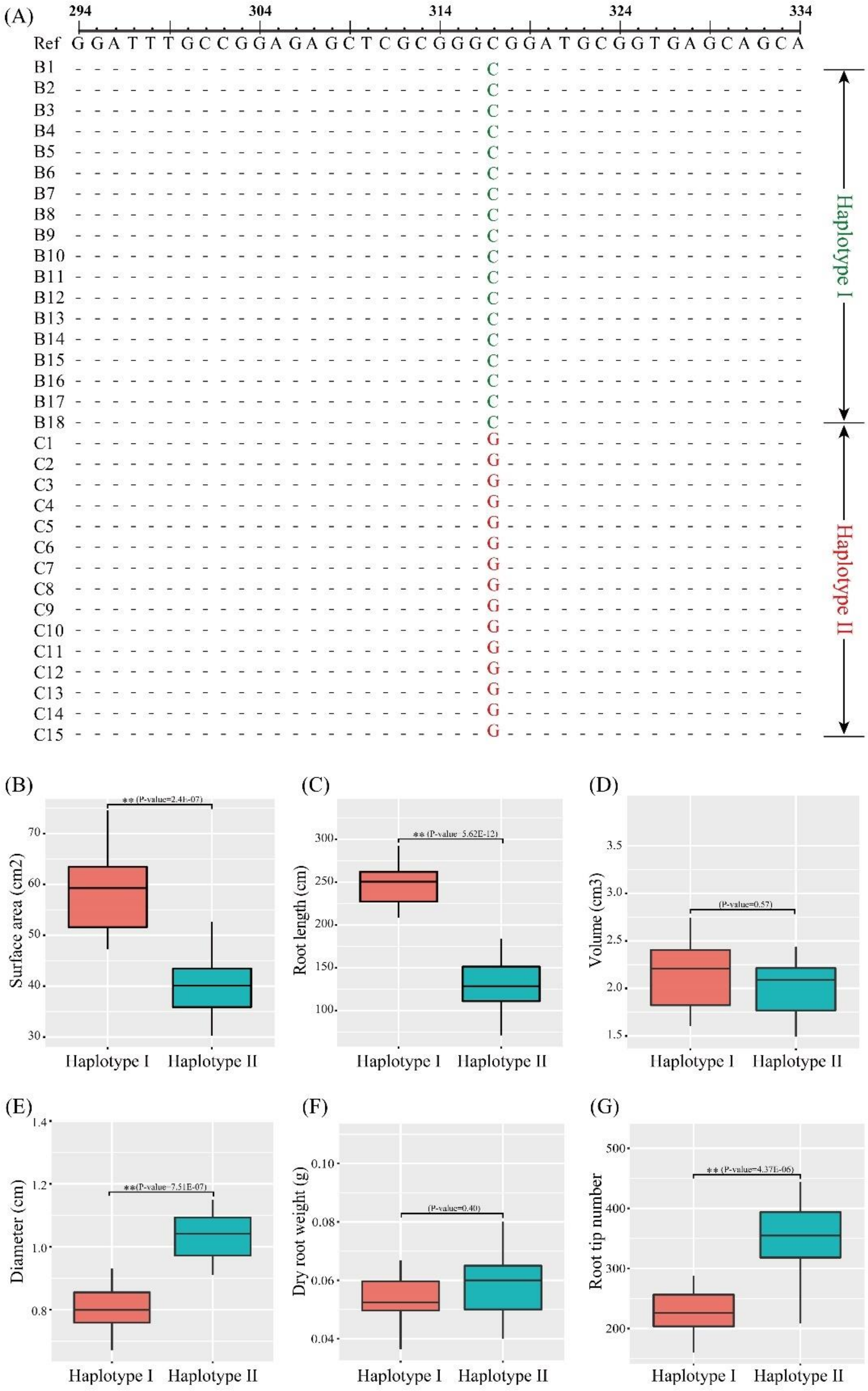

Table S1. Summary and mapping statistics of 33 wild barleys re-sequenced genome.

Table S2. Distribution of INDELs within various genomic regions of 33 wild barley accessions

Table S3. Genes in selected regions and GO and KEGG enrichment analysis.

Table S4. Haplotypes of selected 6 genes.

Table S5. KEGG and GO enrichment analysis of DEGs in leaf samples.

Table S6. KEGG and GO enrichment analysis of DEGs in root samples.

Table S7. Overlap of select genes and highly expressed genes in the leaves and roots of the basalt population.

Table S8. Overlap of selected genes and highly expressed gene in leaves and roots of the chalk population.

Table S9. Overlap of selected genes and DEGs in leaves of the basalt population.

Table S10. Overlap of selected genes and DEGs in leaves of the chalk population.

Table S11. Overlap of selected genes and DEGs in roots of the basalt population.

Table S12. Overlap of selected genes and DEGs in roots of the chalk population.

Table S13. GO enrichment analysis of DEGs in leaves of the basalt population.

Table S14. GO enrichment analysis of DEGs in leaves of the chalk population.

Table S15. GO enrichment analysis of DEGs in roots of the basalt population.

Table S16. GO enrichment analysis of DEGs in roots of the chalk population.
